# The training of midwives to perform obstetric ultrasound scan in Africa for task shifting and extension of scope of practice: a scoping review

**DOI:** 10.1186/s12909-023-04647-w

**Published:** 2023-10-12

**Authors:** Sanele Lukhele, Fhumulani Mavis Mulaudzi, Nombulelo Sepeng, Khathutshelo Netshisaulu, Roinah Nkhensani Ngunyulu, Maurine Musie, Rafiat Anokwuru

**Affiliations:** 1 Department of Nursing, University of Johannesburg, Johannesburg, Republic of South Africa; 2 Department of Nursing, University of Pretoria, Pretoria, Republic of South Africa; 3 Department of Advanced Nursing Science, University of Venda, Thohoyandou, Republic of South Africa; 4 Department of Maternal and Child Health, Ilishan School of Nursing, Babcock University, Remo, Ogun State Nigeria

**Keywords:** Midwifery education, Screening and diagnostic tests, Antenatal care, Interprofessional collaboration, Obstetric ultrasound scan, Task shifting, Extension of scope of practice

## Abstract

**Introduction:**

Ultrasound scan is one of the essential assessments that is crucial in the early identification of health risks during antenatal care. Its accessibility to women in low-and middle-income countries remains a serious challenge because ultrasound scans are not within the scope of practice for midwives. However, task shifting and extension of scope of practice aim to train midwives to assess pregnant women through an ultrasound scan. This paper aims to report the findings of a scoping review on the training of midwives to perform obstetric ultrasound scans in Africa.

**Methods:**

The 6-step iterative framework for scoping reviews by Arksey and O’Malley was used to determine the extent of qualitative and quantitative evidence available on the training of midwives on obstetric ultrasound scans, which includes specifying the research question, identifying relevant studies, selecting studies, extracting and charting data, collating, summarising, and synthesising and reporting findings.

**Results:**

A total of 12 articles from eight African countries were included in this scoping review. Three main themes and 13 sub-themes emerged and they are: obstetric ultrasound scan training, challenges experienced by midwives from task shifting and extension of scope of practice regarding obstetric ultrasound scan, and the value of task shifting and extension of scope of practice regarding obstetric ultrasound scan to midwives.

**Discussion:**

Despite the available evidence that the training of midwives on obstetric ultrasound scans is essential to ensure the accessibility of quality antenatal health services, the training of midwives on obstetric ultrasound scans in some African countries remains a serious challenge. It is evident from this scoping review results that there is a need for African countries to incorporate obstetric ultrasound scans as part of the scope of practice of midwives. Task shifting necessitates prioritising the training of midwives on the use of obstetric ultrasound scans as one of the steps towards the achievement of the United Nations Sustainable Development Goal number 3 targets by 2030.

## Introduction

Sustainable Development Goal number 3 (SDG) aims to achieve universal health coverage and access to essential healthcare services by 2030 [1]. In low and middle-income countries (LMIC), progress towards achieving this goal has been a major issue despite the availability of healthcare interventions [2]. Assessment of pregnant women with obstetric ultrasound scans by midwives may assist in the early identification of abnormalities, interventions and provision of appropriate treatments. An ultrasound scan is one of the health care procedures that women from LMIC do not have access to because it is mostly performed by other health care professionals (HCPs) in highly specialised areas except for midwives. Although this issue is of concern in LMIC, previous studies indicated that many women in low resource settings especially in sub-Saharan Africa will go through pregnancy without the benefit of even a single ultrasound examination [3].

In response, African countries such as Zambia, Uganda, Kenya, Tanzania, Ethiopia and Liberia have already started training nurses and midwives on the use of obstetric ultrasound scans [4–8]. The importance of competent midwives cannot be overstated in Sub-Saharan Africa, where maternal and neonatal outcomes continue to be poor and health delivery systems are failing [9]. However, the progress for training midwives on ultrasound in South Africa has been sluggish. This is contrary to the latest Guidelines for Maternity Care in South Africa [10] that states that for early diagnosis of the complications of pregnancy, all pregnant women should have access to at least one ultrasound scan between 18–20 weeks of pregnancy. This is a challenge because ordinarily, a basic obstetric ultrasound scan is only done by obstetricians and ultra-sonographers. Due to capacity constraints, the guidelines for maternity care in South Africa advocate for accredited midwives, who have undergone basic ultrasound training, to perform obstetric ultrasound scans as part of task shifting and extension of scope of practice. South African midwives have largely relied on their clinical expertise such as abdominal palpation and history taking to assess and confirm the foetal lie, foetal position, presentation, number of foetuses, foetal size; gestational age, and amniotic fluid volume.

While authorisation by the South African National Department of Health for midwives to perform obstetric ultrasound scans is encouraging, midwifery training in obstetric ultrasound scans in South Africa is extremely rare [8, 9]. Ultrasound training is not part of the basic and postgraduate diploma programmes in the midwifery curriculum of South Africa. Secondly, an obstetric ultrasound scan is not part of the scope of practice for midwives. Consequently, the midwives’ illiteracy levels regarding the obstetric ultrasound scan may lead to difficulty in confirming the gestational age of women who are unsure of their last menstrual period, late diagnosis of multiple pregnancies and the misdiagnosis of foetal and pregnancy-related abnormalities [11]. Consequently, task shifting and extension of the scope of practice for midwives regarding obstetric ultrasound scan is essential to achieving Sustainable Development Goal (SDG) target 3.7 which aims to ensure universal access to sexual and reproductive healthcare services. Nurses and midwives make up over 50% of the combined healthcare workers, hence they are central to achieving SDG 3 [2]. Upskilling of midwives results in better screening, diagnosis, and management of pregnant women. The task shifting of obstetric ultrasound scans to midwives is an innovative solution that ensures an affordable and accessible point-of-care ultrasound (POCUS) [12]. The World Health Organization (WHO) [13] supports task shifting and extension of the scope of practice as a strategy for strengthening and expanding the health workforce. To our knowledge, there is a minimal qualitative and quantitative evidence of a scoping review to determine the extent and availability of information on task shifting and extension of scope of practice regarding obstetric ultrasound scans to midwives in Africa.

## Research method

A scoping review was used to determine the extent of the qualitative and quantitative evidence available on task shifting and extension of scope of practice regarding obstetric ultrasound scans to midwives. This evidence was then presented as a visual map. Arksey and O’Malley’s 6-step iterative framework for scoping reviews was used [14]. The six steps are to specify the research question; identify relevant studies; select studies; extract and chart data; collate, summarize and synthesise, and report findings.

### Review question

The researchers searched the literature using two central questions which are:

- What is the available evidence on the training of midwives in obstetric ultrasound scans in Africa?
- What are the findings on the impact of task shifting and extension of scope of practice regarding obstetric ultrasound scans on midwives in Africa?

### Search strategy

The literature search was conducted between September and October 2022 using PubMed, Scopus, Science Direct, EbscoHost, and CINAHL databases. Search terms included (antenatal, prenatal, prebirth, or pregnancy) AND (ultrasound or sonography or sonogram or ultrasonography) AND (midwife or midwives or midwifery) AND (Africa). The final search was limited to articles published between January 2012 and October 2022, as a preliminary search demonstrated that there were few studies published on this topic before 2012.

### Eligibility criteria for inclusion of publication

Inclusion criteria included articles reporting on the training of obstetric ultrasound by midwives who work across Africa. The exclusion criteria were studies published in a non-English language, studies published before 2012, studies that evaluated the use of obstetric ultrasound scans by members of the multidisciplinary team who are not midwives, and studies that presented results from outside Africa.

### Data selection and extraction

**Fig. 1:**
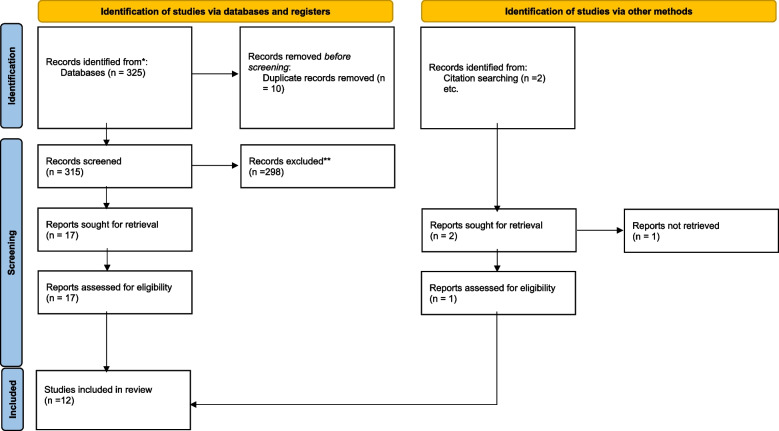
Prisma flow chart

A literature search yielded 325 articles. After removing duplicates, 315 publications were evaluated. Titles and abstracts were screened for inclusion. If inclusion criteria were met, then the full text was retrieved and reviewed. A data abstraction form was developed specifically for this review, including author name, date of publication, population and setting, study aim, study design, evaluation tool, intervention comparator and key outcomes. The abstracts were screened independently by two researchers in the team. The full results were then compared for similarity and consensus. The consensuses’ extracted data were entered into a Microsoft Excel spreadsheet for the organisation. The process and details of the articles included in the final analysis are presented in Fig. 1 and Table 1 respectively. Two researchers independently conducted thematic analyses on the selected publications. The key results with similar meanings were organized into themes and sub-themes. The researchers agreed on the themes and sub-themes to describe the training of midwives in Africa to perform obstetric ultrasound scans for task shifting and scope of practice expansion.

**Table 1:** Summary of articles included in the scoping review

Author, date	Population and setting	Study aim	Study design, evaluation tool	Intervention comparator	Key outcomes
Swanson, et al. [15]. 2014 Uganda	• Midwives • *n* = 14 • 6 Rural health centres	To investigate the diagnostic impact of limited screening obstetric ultrasound To compare how the results of these screening ultrasound performed by midwives corrects the diagnosis when compared with the midwives’ initial clinical assessment	Quantitative	6 weeks course Training methodology utilized lectures, small-group tutorials, audio-visual materials and supervised clinical scanning A logbook was provided to each midwife	• The sensitivity for detecting non-cephalic presentation at 36 weeks or later was 100% (CI: 85 to 100%) • Multiple gestations were often missed before 32 weeks • Limited obstetric ultrasound used as a screening and diagnostic tool by midwives improves the accuracy of diagnosis when compared with the clinical exam • The correction in diagnosis occurred in 6.7 to 7.4% of the obstetric patients screened by midwives by conservative standards, and 11 to 12% of the time with more inclusive standards.
Bentley, Hexom, and Nelson [8] 2015 Liberia	Midwives *n* = 31 Obstetrics Labour and Delivery Outpatient Department at the John F. Kennedy Hospital	To evaluate the effectiveness of a 1-week course for midwives in obstetric ultrasound at a teaching hospital in Monrovia, Liberia	Quantitative prospective study Pre-observational and post-observational assessment	1-week obstetric ultrasound curriculum objective structured clinical examination (OSCE) assessed the participants’ ability to identify the foetal presentation, foetal heart rate, number of foetuses, pregnancy dating, and the placental location Reassessment was done at a 1-year follow-up when the post-test (1-year post-test), post-survey (1-year post-survey), and OSCE (1-year OSCE) were repeated	• There was a significant increase between pre-test scores and immediate and 1-year post-test scores (36.6% versus 90% and 66%, respectively; *P* < .001) • There was no significant difference between immediate and 1-year post-test scores (90% versus 66%; *P* > .05) • The average overall comfort level using ultrasound increased significantly from presurvey to immediate post-survey scores and remained significantly higher at the 1-year post-survey
Holmlund, et al. [16] 2017 Rwanda	Midwives *n* = 23, all female age range: 25–47	To explore the experiences and views of the role of obstetric ultrasound in relation to clinical management, including ethical aspects of midwives from Rwanda	Qualitative Focus group interviews	An interview guide was used for the study	• Unequal distribution of obstetric ultrasound services across the country results in inequity of access to ultrasound • Access to ultrasound is linked to one’s financial status or subscription to medical insurance • Low-risk pregnant women are less likely to be referred for an ultrasound • Lack of training of midwives to perform obstetric ultrasound means that emergency ultrasounds could sometimes not be performed • Informal training of midwives on ultrasound makes them fearful to make clinical decisions based on their findings after the ultrasound examination • Some midwives perceive ultrasonography to not be part of their midwifery duties • Midwives with an interest in learning the skill of ultrasound ask the doctors to teach them practical skill as well as interpreting an image • Participants identified a major need for more healthcare professionals to be performing ultrasound examinations and suggested that it could become part of midwives’ duties • Patients also wish to have an ultrasound done to find out about the sex of the baby to enable preparations for the baby’s arrival • Ultrasound examinations were perceived as important for making decisions regarding whether the treatment of pregnancy complications was possible
Vinayak, et al. [3] 2017 Kenya	Midwives *n* = 3 All have less than 3 years in midwifery	To determine the accuracy of images and reports generated by trained midwives performing basic obstetric ultrasound examinations at our satellite sites The secondary objectives of the project are to (i) implement a teleradiology solution, including protocols to guide communication between sites as a quality control mechanism to review studies from newly trained frontline healthcare providers Identify components of the education and teleradiology model, which needs to be further improved to facilitate ultrasound examinations by inexperienced users	Quantitative	Blended learning approach The lectures were followed by hands-on practical experience, The training period was for 4 weeks and each day began with a 1-h lecture, followed by 6 h of practical hands-on work and ended with another 1-h lecture in the evening The images and reports were reviewed and validated by 2 radiologists with more than 10 y experience in obstetric ultrasound. During this time, the patient was asked to wait for feedback from the main hospital so she could be rescanned if further imaging was required	• The accuracy of the interpretation of images and all corresponding measurements in the report was 99.63% • The turnaround time from the end of the scan to the validation of the report was approximately 15 min • Patients felt that the process was safe, convenient, and reassuring • Patients had a better antenatal visit experience and increased confidence in the delivery of care • More spouses accompanied the mothers for the scans compared with those accompanying them for a routine antenatal clinic visit • All the mothers reported that the scan fostered a stronger bonding between expecting fathers and their babies
Åhmana, et al. [7] 2018 Tanzania	• Midwives, • *n* = 31 • Age: 28–51 • Mean years of experience: 9 • 1 tertiary hospital • 2 regional hospitals	To explore Tanzanian midwives’ experiences and perceptions of the role of obstetric ultrasound in clinical management of pregnancy, and in situations where maternal and foetal health interests’ conflict	Qualitative Focus groups discussions	Thematic interview guide	• Ultrasound improves medical management in complicated pregnancies • Limited ultrasound machines and providers lead to inadequate pregnancy monitoring and diagnosis • Relying on sonographers for diagnosis can delay clinical management
Argaw, et al. [4] 2018 Ethiopia	Midwives, *n* = 24 18 = female, 6 = male, 18–38 years old Experience- 2.5 years- 6.6 years	To explore the experiences and opinions of trained midwives who are providing limited obstetric ultrasound services	Qualitative, descriptive, exploratory In-depth, semi-structured individual interviews	• A 10-day long ultrasound training program • Three monthly coaching sessions Mentors provided immediate feedback for the interpretation of scans • OSCE-based skill test	• Training improved skills and morale • Ultrasound increases midwives’ workload and time-consuming • Unmet patient’s expectations • Increased access to ultrasounds and referral • The community accepted the service • Sustainability depends on funding • Barriers are a lack of adequate infrastructure, power, and equipment • Increased demand for ultrasound is overwhelming for midwives • If the trained person is not on duty or resigns, the services are interrupted
Vinayak and Brownie [12] 2018 Kenya	Midwives, *n* = not specified; hospital	Task sharing project for midwives to fill the gap caused by a shortage of sonographers and grow their competence	Quantitative • Pre-training assessment • In training assessment • End of training assessment • In practice assessment	• 4 weeks training 1-h theory, 6-h practical • Direct Observational work in Practice skills and a written exam was undertaken	• Midwives demonstrated reductions in the time of scanning from 45 to 20 min • Post-delivery accuracy of scans performed by the midwives was 99.63%
Santos, et al. [17] 2020 Uganda	• 3 midwives per phase	To evaluate if an ultrasound-based triage and checklist improved the ability of PHC midwives to correctly diagnose high-risk conditions and appropriately initiate a referral to the district hospital	Quantitative	Phase 1: Intake log Phase 2: Checklist Phase 3: Checklist and ultrasound scan	Ultrasound increased diagnostic suspicion and complications of pregnancy Ultrasound results in an increased incidence of false-positive diagnoses resulting in increasing the burden of care for false positives referred to an already overburdened system
Shah, et al. [6], 2020 Uganda	• Midwives • *n* = 23 • 2 nurses • One Public district hospital • 3 Health centres	An evaluation of a new curriculum for apprentice ultrasound users in Eastern Uganda to diagnosis of high-risk conditions at the point of labour triage using POCUS	Mixed methods Standardized study tools, including a clinical assessment/ultrasound checklist for each woman assessed by the midwives	2-week point of care ultrasound on the identification of 6 complications of pregnancy	Quantitative results • Post-training surveys show that the confidence of the participants improved for measuring foetal heart rate, assessing malpresentation identifying multiple gestations, placenta previa and oligohydramnios • Midwives’ confidence levels increased for measuring gestational age • Of the 25 who took their OSCE exams, 10/11 from the district hospital and 12/14 from the health centers passed (> 80%) on their first attempt with an average score of 90.4% • The first attempt OSCEs revealed 92% of midwives (23/25) could correctly measure foetal heart rate, 100% (25/25) were able to identify the foetal position, 92% (23/25) could correctly follow the placental edge to the most inferior border to assess for Previa, and 84% (21/25) were able to identify and measure the deepest verical pocket (DVP) of amniotic fluid • Estimated Gestitional Age (EGA) measures proved more difficult: correct measures for Head circumference, 52% (13/25), Biparietal Diameter56% (14/25), Femur Length 48% (12/25), and Transcranial doppler 12% (3/25) were performed during the initial post course OSCE exams • Correct use of the aggregate report for estimated gestational Age was performed 92% (23/25) of the time • For each part of the POCUS protocol, quality and measurement ability over time improved among both training cohorts Qualitative Results • Trainees at both the district health and health centre felt having written materials were useful • Trainees valued practical exposure • Mentoring visits enhance the trainee’s skills • Awarding certificates at the end of the training is important • There was a desire that gender identification could have been a part of the training to satisfy mothers’ desires
Vinayak, et, al [11]. 2021	Midwives Kenya 1 Tertiary Hospital, 3 outreach locations	The task-sharing intervention was designed to deliver a high-quality POCUS with measurable changes in Human Resources for Health (HRH) capacity building and proven accuracy in the detection of a range of pregnancy-related anomalies. This paper reports on a curriculum that was developed and implemented and includes evidence of the effectiveness of the program. The innovation involves the delivery of POCUS services by an alternate cadre of health workers, specifically, midwives working under the supervision of a specialist radiology team	Quantitative	4 weeks of training including an e-learning module Written and practical assessments	Midwives demonstrated an image interpretation accuracy of 99.63%
Abdul-Mumin [18], et al. 2022 Ghana	Records in the registry of neonates admitted with congenital anomalies Tamale Teaching Hospital	To determine the proportion of congenital anomalies among neonates admitted to the Tamale Teaching Hospital NICU that can be expected to be detected by prenatal ultrasound screening by Ghana Health Service (GHS) course-	• Quantitative retrospective chart review	2-week training using the Ghana Health Service midwifery training to determine the foetal number, and gestational age, for placental conditions, measure amniotic fluid levels and identify internal and external foetal structures–including detect anomalies abstraction template was used to record maternal and neonatal data, including prenatal ultrasound and maternal characteristics from ANC cards, and delivery history from inpatient notes	• Data from the 2011–2015 chart review and 2016 registry data, revealed that congenital abnormalities are identifiable on ultrasound between 14–23 weeks gestation, • 47% and 43% of congenital anomalies are detectable on ultrasound by GHS course-trained midwives.43–47% of neonates presenting to the NICU presented with readily identifiable under “optimal” conditions and by GHS course-trained midwives, • Midwives can potentially prenatally identify another one-third of the anomalies
Mashamba, et al. [19] 2022 South Africa	Midwives *n* = 10 South Africa 35 health facilities, 10 Midwife-Obstetric Units (MOU) and 15 Community Health Centres (CHC)	A report on evaluation findings during a 12-month pilot period where trained Advance Midwives provided ultrasound services in 35 Midwife-Obstetric Units across the Gauteng Province of South Africa	Quantitative A retrospective descriptive evaluation of the recorded data	3 months of Limited Obstetrics Ultrasound Training organised by General Electric Primary and Referral Care Division Each midwife had to log her entry into the Ultrasound Scan Logbooks	• The midwives provided ultrasound services to 97% of women attending antenatal care at the clinics that were involved in the study. Only 3% of patients came in having already done a scan • Foetal malpresentation, multiple pregnancies and low-lying • Placenta where the commonest anomaly is detected. Consistent with the pattern of ANC attendance, we observed that 60% of complication was identified early < 28 weeks of gestation • A 3% increase in overall referral out based on ultrasound findings for both facility types combined was observed eight months after the implementation of the program • Access to first ultrasound was at 97%, 25% of whom were already in their third trimester

## Results

Nineteen full-text articles were reviewed, and 12 articles which met the inclusion criteria were included in the scoping review. The extracted articles emanate from Kenya [3, 12], Ethiopia [4], Rwanda [16], Tanzania [7], Ghana [18], Uganda [6, 15, 17], Liberia [8] and South Africa [19]. Two studies employed the quantitative pre-test, and post-test design [8, 12]. Four studies applied a retrospective descriptive evaluation of records such as midwives’ logbooks or patient records [15, 17–19]. Three studies followed a qualitative design [4, 7, 16]. One study followed a mixed methods design [6]. Two studies [7, 16] were part of multi-national cross-country studies.

Three themes that were supported by thirteen sub-themes emerged from the final analysis of the articles (scoping review) as follows: obstetric ultrasound scan training, challenges experienced by midwives resulting from task shifting and extension of scope of practice regarding obstetric ultrasound scans, and the value of task shifting and extension of scope of practice of midwives regarding obstetric ultrasound scan to midwives.

### Obstetric ultrasound scan training

#### Mode of training

The literature reveals that there is no standard duration for obstetric ultrasound scan training for midwives. The training duration across different countries ranges between 1 week [8] and 3 months [19]. The results of this review revealed that the training was provided to midwives by different HCPs such as radiologists, family physicians, emergency physicians and or obstetricians [4, 6, 11]. In some instances, training programmes made use of sonographers and radiographers [15]. Despite this, the results of the current review revealed that three midwives from Uganda were trained to be trainers of obstetric ultrasound scan [6].

Most of the training programmes in the current review comprised lectures, small group tutorials, audio-visual materials and supervised clinical scanning was done using face-to-face lecturer method [3, 4, 6, 8, 12, 15, 18]. However, a study done in Kenya incorporated an e-learning module into their curriculum when providing training to midwives [11].

#### Curriculum content for the obstetric ultrasound scan training for midwives

There were similarities and differences in the curriculum content for obstetric ultrasound training among midwives. The most common standard curriculum for obstetric ultrasound scan training across the different countries in the current review consisted of normal anatomy, measuring foetal biometry, estimating amniotic fluid level and determination of gestational age. The curriculum also had the content to teach midwives how to detect foetal cardiac activity, foetal position, number of foetuses, placental location and diagnosis of foetal growth disorder [4, 6, 17, 19]. A study conducted in Uganda had the curriculum content to teach midwives to diagnose preterm labour, multiple gestation, oligohydramnios, placenta praevia, malpresentation and abnormal foetal heart rate using ultrasound among pregnant women in labour [17]. In addition, the study conducted in Kenya had the curriculum content to teach midwives about ultrasound physics, and instrumentation [10]. In Uganda, they had a curriculum content to teach midwives about the maintenance of ultrasound equipment [15]. Furthermore, midwives were also equipped with the knowledge of how to diagnose complications of pregnant women in the first trimester [8].

#### Midwives’ assessments following obstetric ultrasound training

Assessment methods included oral and practical competency tests [15]. The results of the post-training assessment using ultrasound revealed that midwives from Liberia had an increased comfort level of using obstetric ultrasound. This was evaluated using the 4-point Likert scale. The confidence scales increased from 1.8 during pre-training assessment to 3.8 after post-training assessment [8]. The same study demonstrated that participants’ OSCE results following the training showed a significant increase and the level of knowledge was maintained even one-year post-training [8]. This demonstrates that the participants continued to use the skill gained even after the training or else the results would have shown attrition after a year.

#### Accuracy of midwives’ interpretation of basic obstetric ultrasound

A study conducted in Uganda demonstrated that midwives were able to diagnose non-cephalic presentations 100% of the time. However, the diagnosis of twin pregnancy before 32 weeks was a challenge during the training of midwives [15]. It was considered that in 7.4% of the time, ultrasound enhanced midwives’ clinical management of patients by correcting their initial diagnosis that they did through clinical assessment [15]. A Kenyan study confirmed that midwives would scan a patient and make a follow-up with the patient postdelivery. Confirmation of findings found that the postdelivery accuracy of the scans was 97.63%. Therefore, this is an indication that midwives are capable of being taught ultrasound successfully [12]

### Challenges experienced by midwives resulting from task shifting and extension of scope of practice regarding obstetric ultrasound scans.

#### Increased workload for midwives

Midwives welcomed the use of ultrasound to promote access to healthcare services and early identification of complications, thereby facilitating timeous management. However, they also raised concerns about increased workload, resulting from task shifting and extension of the scope of practice for midwives [4]. In support of this, the study findings revealed that midwives found it challenging to cope with the existing burden of workload even before task shifting and extension of the scope of practice, hence they suggested that more midwives be provided with the training to ensure a more equitable distribution of workload [12].

#### Patient’s expectations

 The midwives were unable to print the scans for the patients, the patients in turn were disappointed by this [4]. Expectant mothers would also consult with the expectation of having an ultrasound performed to determine the sex of the foetus [7]. Their expectations to know the sex of the baby, in cases where this is not performed voluntarily, pushed them to pretend to be experiencing the absence of foetal movement to undergo a scan. Midwives are unfortunately not trained to attend to this request as their training does not include gender identification [16].

#### Inaccurate ultrasound scan results

The results of this review illustrated that one midwife told the pregnant woman that the results of the scan revealed that the baby was normal. However, the baby was born with a clubbed foot [4]. This finding illustrated that presenting inaccurate ultrasound scan results among pregnant women may lead to escalated litigations against midwives.

#### Shortage of trained midwives to perform obstetric ultrasound scans

There are a limited number of midwives performing obstetric ultrasound scans for pregnant women in these facilities. Resignations of midwives who are trained in obstetric ultrasound further adds to this [4]. This shortage results in the disruption of providing health care services among pregnant women including obstetric ultrasound services. In these instances, some hospitals have relied on radiographers to perform obstetric ultrasound scans among pregnant women. It was also noted by midwives that the shortage of trained people offering ultrasound services was used by non-medically trained professionals for profit purposes resulting in the issuing of inaccurate reports to unsuspecting patients [7]. This is a major concern as it could impede high-risk patients from seeking medical attention in the case that there are abnormal developments about their pregnancy.

#### Lack of adequate infrastructure and consumables

Studies from the current review reported that there is unavailability of private rooms to perform obstetric ultrasound scan [4] Therefore, it was difficult for them to perform obstetric ultrasound scan among pregnant women. The other challenge was the lack of power supply which is regarded as a barrier to providing health care service [4] among patients. Another study reported concern in the lack of consumables to perform obstetric ultrasound scan among pregnant women, such as ultrasound gel, memory sticks, referral forms among others [4].

### Value of task shifting and extension of the scope of practice regarding obstetric ultrasound scans to midwives

#### Improved access to antenatal care

The task shifting and extension of the scope of practice regarding obstetric ultrasound scan to midwives increases access to obstetric ultrasound during pregnancy. This was evidenced by a study done in South Africa which demonstrated that 97% of pregnant women in a particular primary health care facility had received an ultrasound scan during pregnancy and this enhanced the utilization of antenatal care [19]. As regards, increased access to ultrasound, there was a 3% increase in overall referrals based on the ultrasounds which were carried out by the midwives [19]. The other study conducted in Uganda to triage patients reporting labour increased the likeliness of referral for high-risk conditions. This therefore increased the burden of false positives reporting to an already overburdened higher level of care [17].

#### Improved maternal outcomes

The reviewed literature demonstrated that obstetric ultrasound scans improved perinatal outcomes in pregnant women. Midwives considered ultrasound to be a life-saving diagnostic tool, especially for obstetric emergencies such as antepartum haemorrhage [7] because they were able to refer them to a higher level of care timeously.

#### Improved neonatal outcomes

Midwives saw ultrasound training as a key intervention in decreasing maternal and neonatal mortality as they were able to diagnose the location of pregnancy and the lie of the foetus, determine foetal well-being, assess the placental position, and detect gross foetal abnormalities [4, 7]. The assessment of the cervical length through obstetric ultrasound was also seen as an important predictor of preterm labour [18]. Therefore, this practice enabled them to plan whether or not to administer corticosteroids for lung maturity to the foetus [16]. One of the most striking results to emerge from the data was that obstetric ultrasound scan training equipped the midwives to identify congenital abnormalities that are more visible on ultrasound such as gastroschisis, hydrocephalus and spinal bifida [18].

#### Midwives’ readiness and willingness to learn the performance of obstetric ultrasound scan

Midwives advocated for obstetric ultrasound scan to be part of their routine duties [16]. Another study reported that the basic obstetric ultrasound programme results in increased confidence in midwives [4]. Despite this, some of the midwives expressed that obstetric ultrasound scan was the duty of a physician and expressed discomfort in performing obstetric ultrasound citing safety concerns for the foetus [16].

## Discussion

This scoping review focused on 12 studies. The studies found in this review were conducted across 8 African countries which are Kenya [3, 12], Ethiopia [4], Rwanda [16], Tanzania [7], Ghana [18], Uganda [6, 15, 17], Liberia [8] and South Africa [19]. These studies were also conducted from three different levels of care which are primary [6, 15, 17, 19], district [6, 7, 11] and tertiary [7, 8, 11, 18]. The current scoping review demonstrates that countries across Africa are making efforts to train midwives on how to perform obstetric ultrasound scans. The efforts of training midwives in these countries form part of the cumulative strategies of improving access to maternal health care services of pregnant women in African countries.

The results of the current review illustrate that there is no specific duration for providing obstetric ultrasound scan training in different countries. However, the longest training of obstetric ultrasound scans for midwives was 3 months [19]. In these training midwives were taught how to perform basic scans to determine basic information such as gestational age [6, 18, 19], identify obvious congenital abnormalities on ultrasound [6, 18] and identify high-risk features of pregnancy on ultrasound [6, 19]. In contrast midwives who were trained to be on ultrasound scanning for pregnant women maybe regarded as train the trainers while waiting for the development of the curriculum that maybe used to train midwives of ultrasound. Training the trainers may positively assist in boosting their confidence levels further. The purpose of training these midwives was to make sure that they provide continuous mentorship to the midwives who were part of the study cohort. Therefore, this necessitated the need for developing a curriculum that maybe used to train midwives on ultrasound in Africa. However, the competencies for the development of the curriculum and extension of the scope of practice must be regulated by nursing councils regulating the curriculum for midwives based on the competencies that needs of a specific country.

There were both negative and positive impacts on task shifting and extension of the scope of practice regarding obstetric ultrasound scans to midwives. On the negative impact of task shifting and extension of scope of practice regarding obstetric ultrasound scan, midwives stated that performing obstetric ultrasound negatively impacts their workload [4] and they have a low number of trained midwives in ultrasonography [16]. Provision of inaccurate ultrasound scan results to patients, was also identified as a negative factor. The literature review is silent on inaccuracy of results to pregnant women by other health care professionals after performing ultrasound scan. It is therefore important for midwives to explain to pregnant women that they are using a basic scan with limited information to rule out all foetal abnormalities, before the commencement of the procedure. Inadequate infrastructure was also pointed out as a barrier to performance of obstetric ultrasound scans. Interruption of power supply and lack of ultrasound equipment also proved to be a stumbling block for offering obstetric ultrasound scan [4]. However, it might be useful to consider such backup alternatives as generators to avoid interruption of healthcare services. These challenges must be addressed because they have the potential for interrupting the provision of maternal healthcare services and for promoting access to healthcare services.

Despite this, there was a positive impact of task shifting and extension of scope of practice regarding the training of obstetric ultrasound to midwives. Midwives indicated that the obstetric ultrasound scan assists them to identify the risks and complications associated with the women’s pregnancy timeously. Consequently, early referrals of women experiencing pregnancy complications can be done hence reducing maternal and neonatal mortality rates. The findings of this scoping review were consistent with a narrative review on the use of obstetric ultrasound in African countries [20] and another narrative review on achieving universal access to obstetric ultrasound in African countries [21]. Based on the findings of this scoping review, there is a need to empower and train every midwife on how to use obstetric ultrasound in African countries. This can reduce the existing workload among midwives that are already trained. Also, there is a need to incorporate obstetric ultrasound training in both the undergraduate and postgraduate curricula of midwifery training in African countries. In-service training must be done for the midwives that are already in practice to expedite the task shifting and extension of scope of practice regarding obstetric ultrasound training to midwives as they are the first contact and primary caregivers for pregnant women across all levels of care in African countries.

The limitation of this review is that the literature only included studies which were conducted in African countries, therefore, the researchers are unable to generalise the results. Future research should focus on engaging the nursing regulatory bodies in African countries to include obstetric ultrasound as a competency in midwifery curricula.

## Conclusion

Access to obstetric ultrasound training for women in African countries necessitates task shifting and extension of scope of practice to enable midwives to be trained in large numbers on the use of obstetric ultrasound. Training midwives on the use of obstetric ultrasound scans are essential in Africa to ensure universal access to obstetric ultrasound. Midwives that are trained must be used as mentors to teach their colleagues on how to perform obstetric ultrasound scans when caring for pregnant women. More African countries need to be cautious about training a maximum number of midwives to ensure the sustainability of ultrasound service when it is finally rolled out. Each healthcare facility must have a backup plan for power cuts to avoid interrupting services needed by pregnant women. Nurse managers must budget and purchase consumables needed to conduct obstetric ultrasound scans. Ultrasound services can promote successful task shifting and extension of scope of practice regarding obstetric ultrasound scans to midwives in Africa.

## Data Availability

All data generated or analysed during this study are included in this published article.

## Glossary

Task shifting: is a global recommendation from World Health Organization that involves the coherent redistribution of tasks to individuals within the healthcare team with fewer qualifications that conventionally were not within their scope of work [22].
Extension of scope of practice: The South African Nursing Council (SANC) defines scope of practice of a Nurse or Midwife as the” knowledge, practices and attitudes required to fulfil a professional role” [23]. Scopes of Practice can be used as a reference when writing standards for educational programmes and to provide guidelines for workers. It usually does not include a complete list of what a professional is allowed to do. Saxon, Gray & Oprescu [24]add that extended or expanding scope of practice is operationalised as permitting healthcare workers additional roles to improve patient access to timely health care through accessing specialized training outside the traditional scope of their discipline.
Obstetric ultrasound scan: it is a non-invasive imaging, and accurate method to clinically evaluate and confirm if a woman’s pregnancy is viable and the fetus is growing in intrauterine environment throughout the woman’s pregnancy and confirm gestational age [25]

## References

[1] Department of Economic and Social Affairs Sustainable Development. https://sdgs.un.org/goals/goal3. Accessed 10 Oct 2022.

[2] Abrokwa SK, Ruby LC, Heuvelings CC, Bélard S (2022). Task shifting for point of care ultrasound in primary healthcare in low-and middle-income countries-a systematic review. EClinicalMedicine.

[3] Vinayak S, Sande J, Nisenbaum H, Nolsøe CP (2017). Training midwives to perform basic obstetric point-of-care ultrasound in rural areas using a tablet platform and mobile phone transmission technology—A WFUMB COE project. Ultrasound Med Biol.

[4] Argaw MD, Abawollo HS, Tsegaye ZT, Beshir IA, Damte HD, Mengesha BT, Gebremedhin ZK, Heyi AF, Guteta AA, Mamo TT (2022). Experiences of midwives on Vscan limited obstetric ultrasound use: a qualitative exploratory study. BMC Pregnancy Childbirth.

[5] Kimberly HH, Murray A, Mennicke M, Liteplo A, Lew J, Bohan JS, Tyer-Viola L, Ahn R, Burke T, Noble VE (2010). Focused maternal ultrasound by midwives in rural Zambia. Ultrasound Med Biol.

[6] Shah S, Santos N, Kisa R, Mike Maxwell O, Mulowooza J, Walker D, Muruganandan KM (2020). Efficacy of an ultrasound training program for nurse midwives to assess high-risk conditions at labor triage in rural Uganda. PLoS ONE.

[7] Åhman A, Edvardsson K, Kidanto HL, Ngarina M, Small R, Mogren I (2018). ‘Without ultrasound you can’t reach the best decision’–Midwives’ experiences and views of the role of ultrasound in maternity care in Dar Es Salaam Tanzania. Sexual Reprod Healthcare.

[8] Bentley S, Hexom B, Nelson BP (2015). Evaluation of an obstetric ultrasound curriculum for midwives in Liberia. J Ultrasound Med.

[9] United Nations Population Fund, International Confederation of Midwives, World Health Organization. State of the world’s midwifery 2021. New York: United Nations Population Fund; 2021.

[10] National Department of Health (2015). Guidelines for Maternal Care in South Africa: A manual for clinics, community health centres and district hospitals, vol.

[11] Vinayak S, Temmerman M, Villeirs G, Brownie SM (2021). A curriculum model for multidisciplinary training of midwife sonographers in a low resource setting. J Multidiscip Healthc.

[12] Vinayak S, Brownie S (2018). Collaborative task-sharing to enhance the point-of-care ultrasound (POCUS) access among expectant women in Kenya: the role of midwife sonographers. J Interprof Care.

[13] World Health Organization (2007). Task shifting: rational redistribution of tasks among health workforce teams: global recommendations and guidelines.

[14] Schultz A, Goertzen L, Rothney J, Wener P, Enns J, Halas G, Katz A (2018). A scoping approach to systematically review published reviews: Adaptations and recommendations. Res Synth Methods.

[15] Swanson J, Kawooya M, Swanson D, Hippe D, Dungu-Matovu P, Nathan R (2014). The diagnostic impact of limited, screening obstetric ultrasound when performed by midwives in rural Uganda. J Perinatol.

[16] Holmlund S, Ntaganira J, Edvardsson K, Lan PT, Semasaka Sengoma JP, Åhman A, Small R, Mogren I (2017). Improved maternity care if midwives learn to perform ultrasound: a qualitative study of Rwandan midwives’ experiences and views of obstetric ultrasound. Glob Health Action.

[17] Santos N, Mulowooza J, Isabirye N, Inhensiko I, Sloan NL, Shah S, Butrick E, Waiswa P, Walker D (2021). Effect of a labor triage checklist and ultrasound on obstetric referral at three primary health centers in Eastern Uganda. Int J Gynecol Obstet.

[18] Abdul-Mumin A, Rotkis LN, Gumanga S, Fay EE, Denno DM (2022). Could ultrasound midwifery training increase antenatal detection of congenital anomalies in Ghana?. PLoS ONE.

[19] Mashamba T, Eyo A, Masilela S, Busakwe A, Towobola O (2022). Limited Obstetrics Ultrasound in Primary Healthcare Delivery; Outputs for Strategic Considerations: A Review of Pilot Study in Gauteng Province, South Africa. Journal of Gynecology and Obstetrics.

[20] Kim ET, Singh K, Moran A, Armbruster D, Kozuki N (2018). Obstetric ultrasound use in low and middle income countries: a narrative review. Reprod Health.

[21] Luntsi G, Ugwu A, Nkubli F, Emmanuel R, Ochie K, Nwobi C (2021). Achieving universal access to obstetric ultrasound in resource constrained settings: a narrative review. Radiography.

[22] Leong SL, Teoh SL, Fun WH, Lee SWH (2021). Task shifting in primary care to tackle healthcare worker shortages: An umbrella review. European Journal of General Practice.

[23] Republic of South Africa. Nursing Act No. 33 of 2005. Pretoria: Government Printer; 2005.

[24] Saxon RL, Gray MA, Oprescu FI. Extended roles for allied health professionals: an updated systematic review of the evidence. J Multidisciplinary Healthcare. 2014;7:479–488. 10.2147/JMDH.S66746 PMC4206389 25342909

[25] Recker F, Weber E, Strizek B, Gembruch U, Westerway SC, Dietrich CF (2021). Point-of-care ultrasound in obstetrics and gynecology. Arch Gynecol Obstet.

